# Intravenous Brivaracetam in the Management of Acute Seizures in the Hospital Setting: A Scoping Review

**DOI:** 10.1177/08850666211073598

**Published:** 2022-03-21

**Authors:** Kiwon Lee, Pavel Klein, Prashant Dongre, Eun Jung Choi, Denise H. Rhoney

**Affiliations:** 1 Department of Neurology, Division of Stroke and Critical Care, Rutgers, The State University of New Jersey, 12287 Robert Wood Johnson Medical School, New Brunswick, NJ, USA; 2 Mid-Atlantic Epilepsy and Sleep Center, Bethesda, MD, USA; 3 UCB Pharma, Smyrna, GA, USA; 4 15521 UNC Eshelman School of Pharmacy, University of North Carolina at Chapel Hill, NC, USA

**Keywords:** acute seizure, antiseizure medication, brivaracetam, critically ill, intensive care unit, intravenous

## Abstract

**Background:**

Clinical considerations for drug treatment of acute seizures involve variables such as safety, tolerability, drug-drug interactions, dosage, route of administration, and alterations in pharmacokinetics because of critical illness. Therapy options that are easily and quickly administered without dilution, well tolerated, and effective are needed for the treatment of acute seizures. The objective of this review is to focus on the clinical considerations relating to the use of intravenous brivaracetam (IV BRV) for the treatment of acute seizures in the hospital, focusing on critically ill patients.

**Methods:**

This was a scoping literature review of PubMed from inception to April 13, 2021, and search of the American Academy of Neurology (AAN) 2021 Annual Meeting website for English language publications/conference abstracts reporting the results of IV BRV use in hospitalized patients, particularly in the critical care setting. Outcomes of interest relating to the clinical pharmacology, safety, tolerability, efficacy, and effectiveness of IV BRV were reviewed and are discussed.

**Results:**

Twelve studies were included for analysis. One study showed that plasma concentrations of IV BRV 15 min after the first dose were similar between patients receiving IV BRV as bolus or infusion. IV BRV was generally well tolerated in patients with acute seizures in the hospital setting, with a low incidence of individual TEAEs classified as behavioral disorders. IV BRV demonstrated efficacy and effectiveness and had a rapid onset, with clinical and electrophysiological improvement in seizures observed within minutes. Although outside of the approved label, findings from several studies suggest that IV BRV reduces seizures and is generally well tolerated in patients with status epilepticus.

**Conclusions:**

IV BRV shows effectiveness, and is generally well tolerated in the management of acute seizures in hospitalized patients where rapid administration is needed, representing a clinically relevant antiseizure medication for potential use in the critical care setting.

## Introduction

### Incidence and Management of Seizures in Hospital and the Critical Care Setting

Hospital-onset seizures occur commonly as new-onset seizures, and are likely to be acute symptomatic in origin.^1^ The reported incidence of seizures, the majority of which are nonconvulsive, in critically ill patients with altered level of consciousness is approximately 20%.^2–4^ Most seizures requiring acute medical care will involve impaired awareness and responsiveness, with some involving transient loss of consciousness.^5^ A seizure may only be detectable via electroencephalography (EEG), which is pivotal in both diagnosing the seizure and monitoring the response of seizures to treatment in the intensive care unit (ICU).^6^ Seizure activity in critically ill patients can negatively affect patient outcomes, including increased mortality and morbidity, and increased length of hospital stay.^4,7^

Seizures during critical illness are common in patients with pre-existing conditions, such as epilepsy.^8^ Acute symptomatic seizures may also arise secondary to critical illness including new-onset central nervous system insults such as traumatic brain injury, stroke, infection, brain tumor, toxic metabolic encephalopathy, and neurosurgical procedures.^5,9^ In addition, some patients with coronavirus disease 2019 (COVID-19) may develop acute symptomatic seizures because of hypoxia, metabolic and electrolyte imbalances, multiorgan failure, or brain damage.^10,11^ Drug-induced seizures have also been associated with many medications used routinely in critically ill patients, as frequent alterations in renal function may result in more toxic concentrations of drugs at normal dosing.^8^ Status epilepticus is a common neurologic emergency with an overall estimated incidence of 41 to 61 patients per year per 100 000 population, defined as ≥5 min of recurrent seizure activity without recovery between seizures or continuous clinical and/or electrographic seizure activity.^12–14^

### Real-World Challenges of Seizure Management in Critically Ill Patients

Timely and effective management of seizures is essential. There are many real-world challenges in the management of seizures in patients in hospital and the critical care setting.^15^ For example, seizures in critically ill patients may be subtle with little or no clinical correlation which may make diagnosis challenging, and lack of continuous EEG facilities may further impede early diagnosis and treatment. Drug-drug interactions and pathophysiological changes such as pH alterations, fluid shifts, renal dysfunction, and hepatic dysfunction in patients admitted to hospital can lead to pharmacokinetic and pharmacodynamic alterations, resulting in suboptimal or toxic medication outcomes.^15^

Pre-existing or new-onset renal insufficiency or compromise is common in critically ill patients.^15^ Patients with acute ischemic stroke requiring angiography with the use of a contrast agent may also have increased risk of kidney damage through contrast-induced nephropathy.^16,17^ Many critically ill patients may have increased or decreased renal clearance, necessitating dose modifications of renally excreted medications to avoid therapy failure.^15,18–21^ Because it is mostly renally excreted, the therapeutic effects of levetiracetam (LEV) can be compromised by augmented renal clearance in critically ill patients.^21,22^

Patients in the ICU are at risk of delirium which may lead to behavioral disorders such as agitation and psychosis.^23,24^ Behavioral disorders are even more common in the neuro-ICU where patients may already have pre-existing neurological conditions (including acute brain injuries and neurodegenerative diseases such as dementia).^25–27^ Psychiatric and behavioral adverse events of antiseizure medications (ASMs)^28,29^ could make it even more challenging to manage critically ill patients as they are often compounded by underlying medical and neurologic conditions and effects of medications.

Many critically ill patients have respiratory issues related to underlying disease or drug administration, and protecting the airway is important in this population. Central respiratory drive suppression is common during the use of most benzodiazepines and may require endotracheal intubation of the patient to protect the airway.^14,30,31^ For example, after a loading dose of 4 to 8 mg IV lorazepam, the level of alertness is typically depressed and patients may require intubation, especially if they are experiencing convulsive status epilepticus or nonconvulsive status epilepticus (NCSE).^14^

### Antiseizure Medications

Intravenous (IV) ASMs used for the treatment of acute seizures in patients in hospital include LEV, lorazepam, midazolam, diazepam, lacosamide, sodium valproate, phenobarbital, ketamine, pentobarbital, and fosphenytoin/phenytoin.^12,32^ In the European Union, United States, and other regions globally, LEV is indicated for the treatment of focal seizures with or without secondary generalization, myoclonic seizures in patients with juvenile myoclonic epilepsy, and primary generalized tonic-clonic seizures (PGTCS) in patients with idiopathic generalized epilepsy.^33–35^ LEV has been increasingly used at many centers to manage seizures such as early posttraumatic seizures in the hospital and critical care setting, including in patients with acute traumatic brain injury, aneurysmal subarachnoid hemorrhage, arteriovenous malformation, and brain tumor.^36–38^ For continuity of care, patients that have been started on LEV in the hospital may be switched from IV LEV to oral LEV.

**Figure 1.**
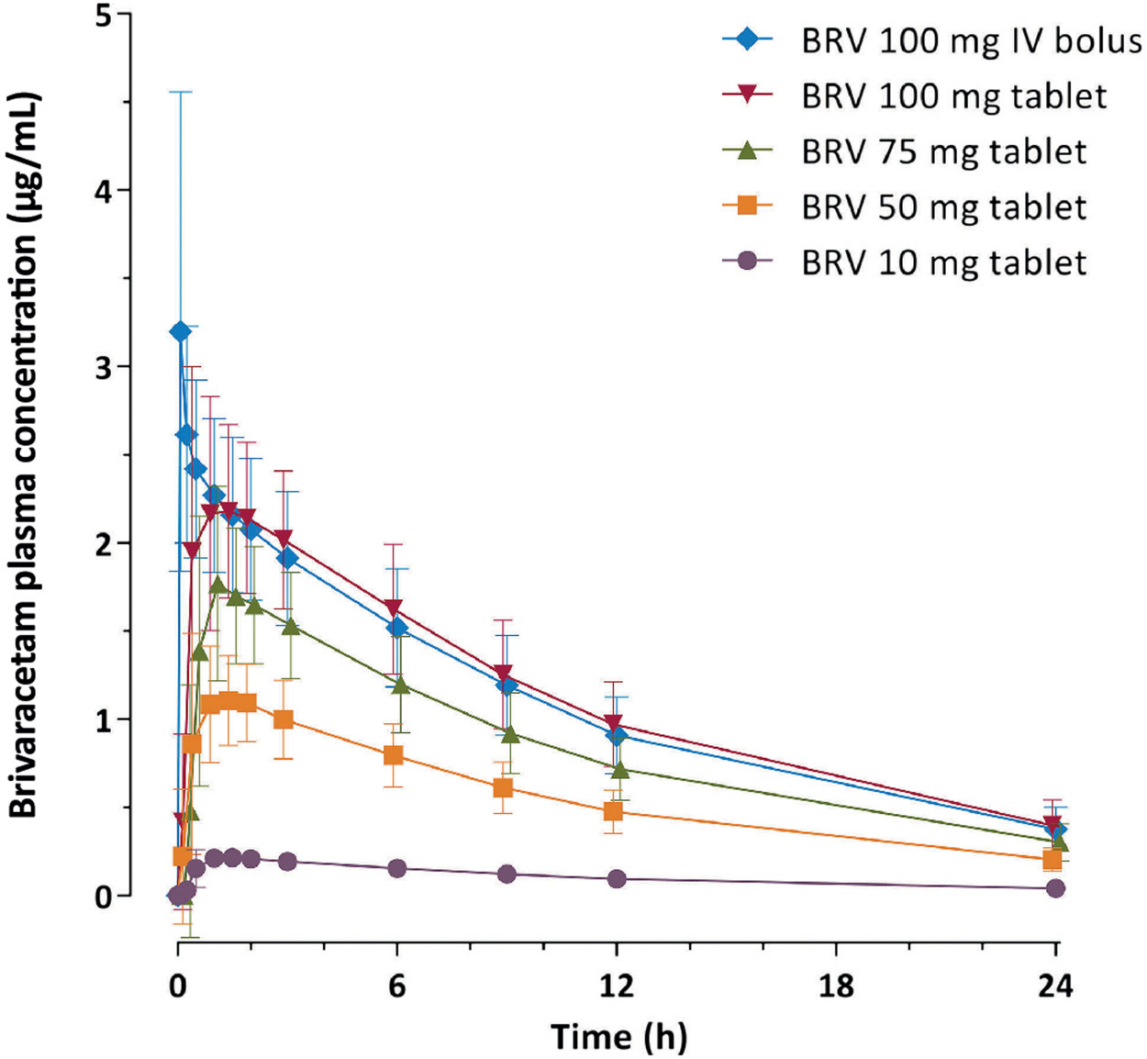
Arithmetic mean (standard deviation) of single-dose brivaracetam plasma concentration versus time profiles during 24 h post dose (pharmacokinetic-per protocol set). BRV, brivaracetam; IV, intravenous.^45^ Figure by Stockis, et al. 2016, CC BY-NC-ND 4.0.

Brivaracetam (BRV) is a second-generation IV-available racetam ASM and analog of LEV that displays high and selective affinity for synaptic vesicle glycoprotein 2A (SV2A) in the brain, and is believed to reduce neuronal excitability by modulating synaptic transmission.^39–43^ BRV is primarily metabolized by hydrolysis, and secondarily via hydroxylation mediated by the cytochrome P450 2C9 and 2C19 isoforms,^32,41^ has minimal clinically relevant drug-drug interactions, and does not interact with drug transporter enzymes.^44^ The time to maximum plasma concentration (t_max_) of BRV 100 mg IV 2-min bolus is achieved in approximately 5 min (vs. 1 h for oral BRV; Figure 1).^45^ Oral BRV has demonstrated efficacy for the adjunctive treatment of adults with focal seizures in randomized, controlled clinical trials in the outpatient setting.^46–48^ Oral BRV is generally well tolerated with a low overall incidence of treatment-emergent adverse events (TEAEs), and a generally low incidence of behavioral and psychiatric TEAEs, allowing treatment initiation at target dose without titration.^49^ In the European Union, BRV is indicated as adjunctive treatment for focal seizures with or without secondary generalization in patients ≥4 years of age.^50^ In the United States, BRV is indicated as monotherapy and adjunctive therapy for the treatment of focal seizures in patients ≥1 month of age,^51^ and is also approved in multiple other regions globally.

Considering the clinical challenges encountered for the treatment of seizures in patients in the hospital and critical care setting, therapy options that have a rapid onset and are well tolerated and effective in diverse settings are needed. Given that the safety and efficacy of oral BRV have been established in patients with epilepsy in the outpatient setting, it is critical to examine the current state of evidence of IV BRV in the hospital setting. IV BRV has the potential to be used for the treatment of acute seizures in the hospital setting (including the ICU, emergency department, and the epilepsy monitoring unit) because it can be easily administered as an IV 2-min bolus and has rapid penetration across the blood-brain barrier, and because oral BRV has shown efficacy and is well tolerated in patients with focal seizures.^49^ The objective of this scoping review was to evaluate evidence related to the clinical pharmacology, safety, tolerability, efficacy, and effectiveness of IV BRV and highlight its utility for acute seizure management in the hospital setting.

## Methods

The five-stage methodological framework for scoping reviews was utilized.^52^ This scoping review focused on addressing the questions: “What does the current state of evidence suggest related to clinical pharmacology, safety, tolerability, efficacy, and effectiveness of IV BRV for the treatment of acute seizures in the hospital setting? What are the gaps in the literature?” PubMed was searched from inception to April 13, 2021. The search query consisted of terms related to IV brivaracetam, seizure, status epilepticus, epilepsy monitoring unit, intensive care unit, and critically ill (Appendix A). Additional sources were found through a manual search of the reference lists of the PubMed studies and through the American Academy of Neurology (AAN) 2021 Annual Meeting website. Included studies were English language articles or abstracts of randomized controlled trials, case reports, retrospective observational studies, and cohort studies of IV BRV in the hospital setting. Literature/narrative reviews, animal studies, letters to the editor, systematic literature reviews, and pooled analyses were excluded. Outcomes of interest were the pharmacokinetics, pharmacodynamics, safety, tolerability, efficacy, and effectiveness of IV BRV.

## Results

**Figure 2.**
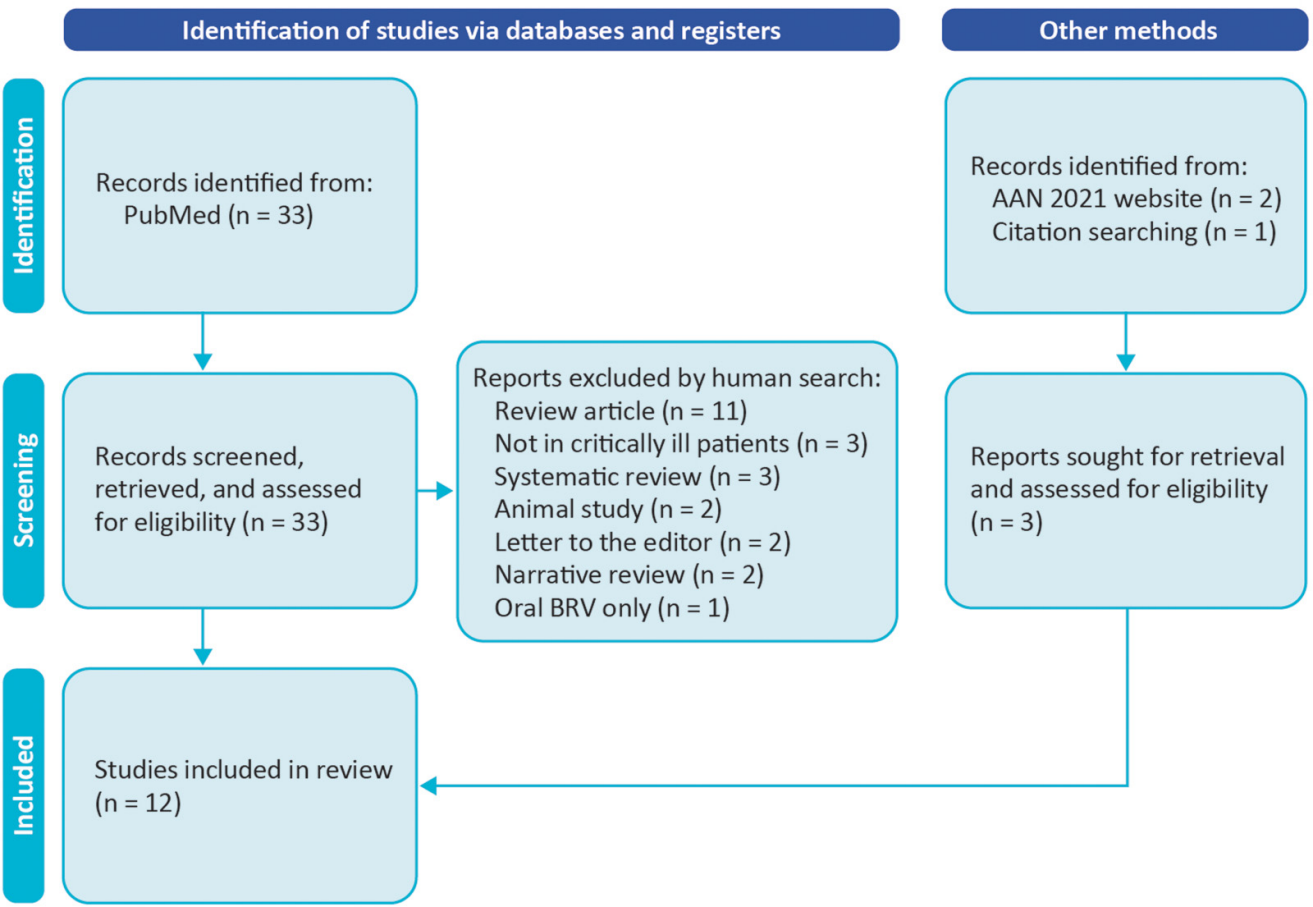
Flow chart describing study selection process. AAN, American Academy of Neurology.

The initial PubMed search yielded 33 studies, the manual search yielded 1 further study, and the AAN website search yielded 2 studies (Figure 2). After applying inclusion and exclusion criteria, 12 studies were included. Table 1 provides the characteristics of the included publications. Table 2 provides the information extracted based upon the selected research questions.

**Table 1.** Characteristics of Included Publications (N = 12).

Characteristic	n
Year of Publication:	
2021	3
2020	3
2019	2
2018	2
2017	1
2016	1
Journals Published:	
*BMJ Case Reports*	1
*CNS Drugs*	1
*Epilepsia*	4
*Epilepsy & Behavior*	2
*Epilepsy Research*	1
*Seizure: European Journal of Epilepsy*	1
Congress:	
*American Academy of Neurology (AAN) 2021 Annual Meeting*	2
Type of Publication:	
Abstract only	2
Full text	10
Study Design:	
Individual case study	1
Prospective, interventional	4
Retrospective, observational	6
Single-center case series	1
WHO Region:	
Americas	4
European	6
Multi-country	2

WHO, World Health Organization.

**Table 2.** Publications Examining Treatment with Intravenous Brivaracetam in Patients in Hospital.

Author, Year, Number of Patients	Average Age of Patients, Years	Country of Study; Setting	Study Design	Dose and Length of Administration (if Available)	Prior or Concomitant ASMs	Main Outcomes and How the Response was Determined	Results		
							Pharmacology	Safety and Tolerability	Efficacy and Effectiveness
Klein et al. 2016^53^ (n = 105)	41.6 (12.2), mean (SD)	17 sites in the US, Poland, Germany, and Czech Republic; the majority (84.8%) were hospital inpatients	Phase 3, multicenter, randomized, 4-arm, parallel-group trial in patients (16-70 years of age) with focal or generalized epilepsy uncontrolled by 1 to 2 ASMs	Run-in, double-blind period: oral BRV 200 mg/day (100 mg BID) or placebo Evaluation, open-label period: IV BRV 200 mg/day (100 mg BID) as 2-min bolus or 15-min infusion	Concomitant ASMs (excluding felbamate and vigabatrin) and vagus nerve stimulation	**Pharmacology:** plasma concentrations of BRV 5 min before and 15 min after IV BRV administration **Safety and tolerability:** seizure counts, adverse events, vital signs, clinical laboratory tests, and 12-lead ECGs	Geometric mean plasma concentrations of IV BRV 15 min after the first dose were similar between patients receiving IV BRV as a bolus or infusion	Incidences of TEAEs and drug-related TEAEs were similar across the treatment arms, and for patients who received IV BRV as a bolus or infusion The most frequent TEAEs were somnolence (29.5%) and dizziness (14.3%) Most TEAEs were mild (63.8%) or moderate (11.4%) in intensity 9.6% and 11.5% of patients reported injection-related TEAEs following bolus and infusion of IV BRV, respectively No serious TEAEs were reported	Decreases in mean seizure frequency from baseline were observed in all patients
Strzelczyk et al. 2017^58^ (n = 11)	64 (21), median (IQR)	Germany; 2 hospitals	Retrospective review of medical records for a cohort of patients with refractory and super-refractory status epilepticus	Initial BRV dosage varied from 50 to 400 mg (median 100 mg, mean 150 mg) titrated up within 1 day to maximum daily doses of 100 to 400 mg (median 200 mg, mean 264 mg)	Multiple ASMs and BZDs were given before and concomitant with BRV, with a direct LEV to BRV switch in 9 patients	**Effectiveness:** cessation of status epilepticus within 24 h of BRV treatment, and absence of further seizures within the next 24 h, as observed by EEG	NA	No serious adverse events were observed in patients during BRV treatment	There was a cessation of status epilepticus in the first 24 h of BRV in 27% of patients Attenuation and resolution of status epilepticus was observed directly by EEG in 1 patient after BRV treatment
Strzelczyk et al. 2018^71^ (n = 2)	**Patient 1:** 28, **Patient 2:** 22	Germany; hospital emergency setting	Multicenter, retrospective cohort study of patients with genetic generalized epilepsies and absence status epilepticus as determined by continuous EEG treated with IV BRV	IV BRV 200 to 300 mg bolus	**Patient 1:** bolus IV LZP up to 4 mg and BRV up to 300 mg, then VPA 1600 mg and LCM 400 mg **Patient 2:** 10 mg intranasal MDZ followed by IV BRV 200 mg bolus, then VPA 1000 mg and LZP 2 mg	**Effectiveness:** cessation of status epilepticus	NA	200 to 300 mg bolus IV BRV was well tolerated for 2 patients with absence status epilepticus	Further ASMs after the administration of IV BRV were required for the cessation of status epilepticus
Kalss et al. 2018^59^ (n = 7)	68 (29-79), median (range)	Austria; neurological emergency room, neurological intensive care unit, or neurological normal ward	Single center, retrospective case analysis of patients with status epilepticus (status epilepticus occurred de novo in 1 patient due to hypoxic brain injury)	Median loading dose was IV BRV 100 mg over 15 min (range: 50-200 mg), titrated up to a median dose of 100 mg/day (range: 100-300 mg)	Median of 4 (range: 2-11) ASMs before BRV	**Tolerability:** adverse events after add-on treatment with BRV **Effectiveness:** treatment response to BRV within 1 h and >24 h, outcome of status epilepticus	NA	No cardiorespiratory adverse events were observed	Improvement in Glasgow Outcome Scale in 86% of patients Immediate clinical and electrophysiological improvement in 29% (2/7) of patients Early electrophysiological cessation of status epilepticus on surface EEG was observed in 43% (3/7) of patients
Aicua-Rapun et al. 2019^60^ (n = 14)	61 (33-80), median (range)	Switzerland; in hospital (outside of the ICU)	Single center, retrospective analysis of patients with status epilepticus treated with IV BRV	Mean loading dose was 171.4 mg and median loading dose 200 mg. Mean maintenance dose was 207.1 mg and median maintenance dose 200 mg	Median 4 ASMs (range: 2-7) before BRV	**Pharmacology:** Clinical response was correlated with measured plasma concentrations of BRV and extrapolated exposure parameters calculated using a population pharmacokinetics model **Effectiveness:** clinical response (defined as BRV being able to resolve status epilepticus without the need of further treatment)	Mean calculated BRV concentrations during the observation period were significantly higher in responders (2.6 mg/L), versus nonresponders (1.8 mg/L); *P* = .01, U test After the loading dose, minimum BRV calculated concentrations were significantly higher in responders (2.2 mg/L) versus nonresponders (1.2 mg/L); *P* = .04, U test	No adverse events were reported related to BRV treatment	50% (7/14) of patients responded to BRV Doses of IV BRV higher than 1.9 mg/kg were associated with a greater likelihood of a response
Santamarina et al. 2019^61^ (n = 43)	56.2 (23.1), mean (SD)	Spain; 7 hospitals (16 patients had treatment in the ICU)	Retrospective multicenter registry review in patients with status epilepticus treated with IV BRV	Median loading dose was 100 mg (range: 25-400 mg). The median infusion rate was 0.18 mg/kg/min (range: 0.08-0.90 mg/kg/min)	In 32 (74.4%) of the 43 patients, BRV was used after failure of other ASMs. In 17 (39.5%) patients, BRV was used even though LEV had already been tried	**Safety:** TEAEs **Effectiveness:** response to treatment of IV BRV as treatment for status epilepticus, monitored clinically and with EEG to verify the disappearance of continuous epileptic activity	NA	6 (14%) patients had TEAEs attributed to BRV; 5 reported somnolence after BRV administration, and 1 experienced seizure worsening BRV was discontinued on 3 occasions because of seizure worsening (n = 1) and excessive somnolence (n = 2)	23 (53.5%) patients responded (cessation of status epilepticus), with 13 (56.5% of responders) having a response within 6 h Patients who responded to BRV were less likely to need IV anesthetics and admission to ICU for this reason
Szaflarski et al. 2020^56^ (n = 45 [IV BRV 100 mg, n = 15; IV BRV 200 mg, n = 15; IV LZP, n = 15])	BRV 100 mg: 43.9 (12.4); BRV 200 mg: 41.6 (16.0); LZP: 40.2 (11.0), mean (SD)	US; epilepsy monitoring units	Phase 2, open-label, randomized, active-control, proof-of-concept trial in patients (18-70 years of age) with epilepsy who experienced seizures requiring acute treatment	IV BRV 100 mg (2-min infusion) or 200 mg (4-min infusion). IV LZP was administered at doses from 1 to 4 mg (median: 1 mg)	0 to 4 ASMs taken at trial entry for 29 patients, ≥5 ASMs taken at trial entry for 1 patient	**Safety:** TEAEs **Efficacy:** time to next seizure (per clinical observation with EEG confirmation) or to rescue medication (representing treatment failure) within 12 h after the end of trial medication administration	NA	TEAEs were reported by 6 (40.0%) patients in the BRV 100 mg group, and 3 (20.0%) patients in the BRV 200 mg group The most common TEAEs with BRV were nausea, dizziness, and headache, consistent with its known safety and tolerability profile	IV BRV (both doses) showed similar efficacy to IV LZP in controlling acute seizure activity in the epilepsy monitoring unit Rescue medication use within 12 h was higher for LZP compared with BRV 100 mg or 200 mg
Reed et al. 2020^55^ (n = 9)	27.8 (18-42), mean (range)	US; comprehensive epilepsy care center	Prospective, randomized, double-blind, 2-period crossover trial in patients with photosensitive epilepsy treated with IV LEV and IV BRV	**Part 1:** IV LEV 1500 mg or IV BRV 100 mg 15-min infusion, then a >14-day washout time before Part 2 **Part 2:** IV LEV 1500 mg or IV BRV 100 mg 5-min infusion Patients were randomized either to LEV first then to BRV, or to BRV first then to LEV	Not reported	**Pharmacodynamic efficacy:** BRV: LEV time ratio to PPR elimination as measured by EEG	NA	Adverse events included mild transient lightheadedness that occurred in 4 BRV-treated patients No severe or serious FDA-reportable adverse events occurred	BRV eliminated PPRs more quickly than LEV did (median 2 vs. 7.5 min, respectively) There was no significant difference in the BRV:LEV time ratio to PPR elimination for the 15-min infusion alone (*P* = .22) or the 5-min infusion alone (*P* = .11) BRV was faster than LEV at eliminating PPR when an outlier patient was excluded for the 15-min infusion (*P* = .0016) When data from the 15- and 5-min IV BRV infusions were combined, PPR elimination was 61% faster with BRV (*P* = .039)
Ammar et al. 2020^62^ (n = 1)	67	US; neuro-ICU	Case study of a patient with focal NCSE refractory to multiple ASMs	Loading dose of IV BRV 200 mg, followed by maintenance dose 100 mg every 12 h	In chronological order: IV LZP (4 mg), IV VPA sodium (loading dose 25 mg/kg, maintenance dose 500 mg every 12 h), IV LEV 1000 mg, IV fosphenytoin (loading dose 20 mg/kg, maintenance dose 150 mg every 8 h), IV LCM (300 mg, maintenance dose 150 mg every 12 h), clobazam 10 mg every 12 h	**Effectiveness:** cessation of NCSE as observed by continuous EEG in a patient with focal-impaired awareness NCSE refractory to LZP, LEV, fosphenytoin, LCM, and VPA	NA	The patient had stable respiratory status while on BRV treatment, with no cardiorespiratory adverse events observed	Definitive clinical and electrographic improvement observed 15 min after the loading dose of IV BRV, with very long periods free of epileptiform activity and resolution of NCSE
Orlandi et al. 2021^63^ (n = 56)	61.9 (19.1), mean (SD)	24 sites in Italy; hospital neurology units	Retrospective, observational, multicenter study on patients with status epilepticus from March 2018 to June 2020	Median loading dose was IV BRV 100 mg (range: 50-250 mg)	BZDs and multiple ASMs such as LEV, phenytoin, phenobarbital, or VPA	**Safety:** TEAEs **Effectiveness:** resolution of status epilepticus (assessed clinically and with EEG)	NA	5 patients reported drowsiness, and 1 had a transitory increase in liver enzymes No severe TEAEs were observed	Status epilepticus resolved in 57% of patients on IV BRV overall, and within the first 6 h of IV BRV administration for 39% of patients 2 patients received IV BRV as first-line treatment to avoid possible respiratory insufficiency, and in these patients status epilepticus resolved within a few minutes Resolution of status epilepticus was faster when IV BRV was administered earlier, both when it was 1 of the first 2 ASMs administered (82% vs. 50%, *P* = .02) and when IV BRV was used within 6 h from onset of status epilepticus (55% vs. 3%, *P* <.001)
Martin et al. 2021^54^ (n = 105 [38 patients were on concomitant LEV treatment])	For the 38 patients were on concomitant LEV treatment: 39.7 (12.6), mean (SD)	US, Czech Republic, Germany, and Poland; for the overall population, the majority (84.8%) were hospital inpatients^53^	Subgroup analysis of a Phase 3, randomized, placebo-controlled trial (Klein et al. 2016^53^^)^ of IV BRV in patients on LEV treatment	Run-in, double-blind period: oral BRV 200 mg/day (100 mg BID) or placebo Evaluation, open-label period: IV BRV 200 mg/day (100 mg BID) as 2-min bolus or 15-min infusion	Concomitant ASMs (excluding felbamate and vigabatrin) and vagus nerve stimulation	**Safety and tolerability:** TEAEs were compared between patients taking IV BRV with concomitant LEV and the overall population during the complete oral and IV BRV study period	NA	TEAEs in patients treated with IV BRV with concomitant LEV were similar to the overall population (TEAEs: 76.3% vs. 76.2%; drug-related TEAEs: 65.8% vs 63.8%, respectively) The most common (≥5%) TEAEs in the overall population of somnolence (29.5%), dizziness (14.3%), headache (6.7%), and fatigue (5.7%), were reported by 26.3%, 10.5%, 10.5%, and 5.3% of patients treated with IV BRV + LEV, respectively The incidence of individual TEAEs classified as psychiatric or potential behavioral disorders was low (≤3%), with no apparent differences between patients treated with IV BRV with concomitant LEV and the overall population	NA
Beaty et al. 2021^57^ 450 (LEV, n = 360; BRV, n = 90) The cohorts were matched 4:1	Adult patients	860 hospitals in the US	Retrospective cohort analysis from 860 US hospitals in the Premier Healthcare Database of adult patients treated with IV BRV or LEV with or without BZDs for seizures within the hospital setting	Not reported	BZDs	**Effectiveness:** ICU admission, all-cause and seizure-related readmission	NA	NA	Patients treated with IV BRV had a significantly lower prevalence of ICU admission compared with patients treated with IV LEV (14.4% vs 24.2%, *P* <.05), and significantly lower 30-day seizure-related readmissions (0% vs 4.2%,*P* <.05) Adjusted odds for ICU admission was 47% lower for patients treated with IV BRV versus IV LEV (*P* <.05)

Abbreviations: ASM, antiseizure medication; BRV, brivaracetam; BZD, benzodiazepine; ECG, electrocardiogram; EEG, electroencephalography; ICU, intensive care unit; IQR, interquartile range; IV, intravenous; LCM, lacosamide; LEV, levetiracetam; LZP, lorazepam; MDZ, midazolam; NA, not available; NCSE, nonconvulsive status epilepticus; PPR, photoparoxysmal response; SD, standard deviation; TEAE, treatment-emergent adverse event; tmax, time to maximum plasma concentration; US, United States; VPA, valproate.

A randomized, Phase 3 trial established the pharmacokinetics, safety, and tolerability of IV BRV (2-min bolus or 15-min infusion) in 105 adult patients with epilepsy, 84.8% of which were inpatients.^53^ Geometric mean plasma concentrations of IV BRV 15 min after the first dose were similar between patients receiving 2-min bolus and 15-min infusion. The incidence of TEAEs with IV BRV was similar whether initiated first or following oral BRV, and the most common TEAEs were somnolence (29.5%) and dizziness (14.3%). Decreases in mean seizure frequency from baseline were observed in all patients. In a subgroup analysis of this Phase 3 trial in patients who were on concomitant LEV treatment, TEAEs in patients treated with IV BRV with concomitant LEV were similar to the overall population, with no new safety signals reported, and a low incidence of individual TEAEs classified as psychiatric or potential behavioral disorders (≤3%).^54^

In a prospective, randomized, double-blind, crossover trial in 9 patients with photosensitive epilepsy in a hospital comprehensive epilepsy care center, IV BRV (100 mg 5- or 15-min infusion) eliminated the electroencephalographic photoparoxysmal response faster than IV LEV (median 2 vs. 7.5 min, respectively).^55^ When data from the 5- and 15-min IV BRV infusions were combined, photoparoxysmal response elimination was 61% faster with BRV (*P* = 0.039).

An open-label, randomized, active-control, Phase 2 trial was conducted in 45 adult patients with epilepsy admitted to the epilepsy monitoring unit who experienced seizures requiring acute treatment.^56^ IV BRV 100 mg (2-min infusion) or 200 mg (4-min infusion) showed similar efficacy to IV lorazepam in controlling acute seizure activity; 80% of patients who received IV BRV were seizure free for over 12 h compared with 60% of patients who received lorazepam, and rescue medication use within 12 h was numerically higher for patients on IV lorazepam. The incidence of TEAEs was generally similar between IV BRV and IV lorazepam.

A retrospective cohort analysis of real-world chargemaster data from 860 United States hospitals in the Premier Healthcare Database was conducted in adult patients treated with IV LEV (n = 360) or IV BRV (n = 90) for seizures within the hospital setting.^57^ Patients treated with IV BRV had a lower prevalence of ICU admission than patients treated with IV LEV (14.4% vs. 24.2%; *P* <.05), as well as lower 30-day seizure-related readmissions (0% vs. 4.2%; *P* <.05), and adjusted odds for ICU admission was 47% lower for patients treated with IV BRV versus IV LEV (*P* <.05). These findings are associations and not evidence of causality, as the order of events could not be determined from the hospital billing data.

Although outside of the approved label, IV BRV has been evaluated in terminating seizures in patients with status epilepticus and refractory status epilepticus. In a retrospective review of medical records for a cohort of 11 patients with refractory and super-refractory status epilepticus, status epilepticus ceased in the first 24 h of BRV treatment in 27% of patients, with attenuation and resolution of status epilepticus observed directly by EEG in 1 patient.^58^ In a retrospective assessment in 7 patients with status epilepticus in the emergency setting, a median loading dose of 100 mg IV BRV resulted in immediate clinical and electrophysiological improvement in 29% of patients, with early electrophysiological cessation of status epilepticus on surface EEG observed in 43% of patients.^59^ The positive treatment response to BRV combined with absence of cardiorespiratory adverse events resulted in no ICU admissions among these patients. Another retrospective analysis in 14 patients treated with IV BRV for status epilepticus reported that doses of IV BRV higher than 1.9 mg/kg were associated with a greater likelihood of resolution of status epilepticus.^60^ A retrospective registry study of 43 patients showed that treatment with IV BRV in both the hospital and ICU ceased status epilepticus in 53.5% of patients, with over half of these patients responding within the first 6 h.^61^ In a case study of a patient with focal NCSE admitted to the neurological ICU who had been unsuccessfully treated with a number of previous IV ASMs (including LEV), definitive clinical and electrographic improvement in NCSE was observed 15 min following 200 mg IV BRV, with very long periods of cessation of epileptiform activity and resolution of NCSE.^62^ A retrospective, observational, multicenter study of 56 patients with status epilepticus in hospital neurology units reported that resolution of status epilepticus was significantly faster when IV BRV was administered earlier, and resolution occurred within a few minutes in 2 patients who received IV BRV as first-line treatment (instead of a benzodiazepine).^63^

Overall, the sample sizes of the included studies were small, and there were gaps in the literature relating to the use of IV BRV for the treatment of acute seizures in the hospital and critical care setting. Very few studies have examined the pharmacology of IV BRV in patients in the hospital setting, and a limited number have assessed the effectiveness and tolerability of IV BRV in the critical care setting. In addition, no data are available on the potentially altered pharmacokinetics of IV BRV in critically ill patients, which are needed to determine accurate dosing of IV BRV in these patients. Finally, further data are required relating to behavioral adverse events and the absence of respiratory depression when patients in hospital are treated with IV BRV.

## Discussion

IV BRV shows efficacy and effectiveness and is generally well tolerated in patients with seizures in the hospital and critical care setting. Although larger well controlled studies are needed, small real-world studies suggest that IV BRV also reduces seizures and is generally well tolerated in patients with status epilepticus, including those requiring treatment in the ICU.

Providing timely and effective management of acute seizures in patients in hospital is essential. Data from healthy volunteers show that therapeutic doses of IV BRV have rapid penetration across the blood-brain barrier and enter the brain faster than IV LEV.^64^ IV BRV acts fast, with 100 mg 2-min bolus achieving a t_max_ in approximately 5 min,^45^ and suppression of epileptiform activity on EEG in a median of 2 min in patients with photosensitive epilepsy.^55^ This translates to the rapid response observed with IV BRV. In numerous studies, resolution of status epilepticus or NCSE was observed directly by clinical observation and EEG within minutes of administration of IV BRV treatment.^58,59,62,63^ A retrospective analysis reported that doses of IV BRV higher than 1.9 mg/kg were associated with a greater likelihood of resolution of status epilepticus, and the authors suggested a loading dose of at least 2 mg/kg in adults for the treatment of status epilepticus.^60^ However, this is outside of the current approved label for BRV, and the dosing for status epilepticus (including weight-based vs. flat dose) has not been formally assessed.

This review has important clinical implications for day-to-day practice in patients in hospital, including in the critical care setting. Although reduced renal clearance of BRV has been observed in patients with renal impairment, dose adjustments are likely not necessary, and this represents an important advantage of BRV in patients with acute renal failure or augmented renal clearance.^65,66^ This is beneficial in the neuro-ICU where iodine contrast is frequently used for computed tomography angiography and digital subtraction angiography, and often patients already have multiple vascular risk factors including hypertension and diabetes.^67^ For example, there may be a patient with impaired renal function, or rising creatinine, for instance in the setting of subarachnoid hemorrhage requiring repeated angiograms for evaluation of vasospasm. If such patients are on a high dose of IV LEV (1 g BID) and high rate of normal saline infusion as part of hypertensive euvolemic therapy, switching to less acidic IV fluids along with switching to IV BRV can be considered. There is also low potential for drug-drug interactions with IV BRV. These clinical considerations are especially important for patients with acute ischemic stroke, intracerebral hemorrhage, aneurysmal subarachnoid hemorrhage with vasospasm with inevitable use of contrast neuroimages and therapeutic digital subtraction angiography.^14^

Patients admitted to the emergency department or ICU often receive LEV or benzodiazepines as an initial treatment for seizures. Psychiatric and behavioral adverse events of ASMs including LEV^28,29,33,43,68^ could make it even more challenging to manage critically ill patients. In a pooled analysis of patients with epilepsy receiving adjunctive oral BRV, psychiatric and behavioral disorders were reported by 11.3% and 4% of patients, respectively (vs. 8.2% and 2.5% with placebo); <1% of patients discontinued due to behavioral adverse events.^69^ In numerous real-world evidence studies, switching from LEV to BRV treatment in patients with epilepsy resulted in resolution of behavioral side effects in approximately two thirds of patients.^43,70–73^ In patients with focal seizures, IV BRV 100 mg BID was well tolerated in patients on LEV treatment, with a low incidence of individual TEAEs classified as psychiatric or potential behavioral disorders.^54^ In addition, IV BRV shows effectiveness and is generally well tolerated in patients in hospital with prior LEV treatment.^58,61–63^

Respiratory depression has not been reported with BRV,^69^ and no cardiorespiratory adverse events were observed in patients treated with IV BRV.^59,62^ This makes IV BRV a good treatment option for patients with acute seizures, as it may not increase the need for endotracheal intubation that is often required for excessive use of IV benzodiazepines or anesthetic medications such as continuous IV lorazepam, midazolam, propofol, or pentobarbital.^14^ Compared with drugs such as lorazepam and propofol, which can cause respiratory depression and paranoia,^74^ IV BRV has the potential to be a viable treatment option without leading to respiratory depression and other negative side effects. More studies are required to provide evidence for the lack of respiratory depression for patients in hospital and the critical care setting treated with IV BRV.

The pharmacokinetics, safety, and tolerability of IV BRV in pediatric patients ≥1 month to <16 years of age with epilepsy was studied in a Phase 2, multicenter, open-label trial.^75^ The results showed that IV BRV given as a 2-min bolus or 15-min infusion was well tolerated at doses up to 200 mg/day, with no new safety concerns identified and no unexpected pharmacokinetic differences observed between infusion groups.

For continuity of care, patients may be switched from IV BRV to oral BRV. Pooled data of Phase 2b/3 and long-term follow-up trials (≥8.0 years) confirmed the safety, tolerability, and efficacy of adjunctive oral BRV for the treatment of focal seizures, with a 5-year retention rate of 54.4%.^49^

## Limitations

There are limitations to this review. Our search may not have been exhaustive as it was only conducted in 1 database (PubMed) and 1 conference website (AAN 2021). Of note, the limited frequency and strength of the existing evidence identified in this review (particularly a number of smaller, retrospective case studies and chart reviews) should be interpreted in context and reflects the difficulty of capturing real-world data for patients during critical seizure events. Larger, more robust studies are needed to confirm the trends summarized here. Currently, BRV has not been included in NCSE management guidelines and not enough comparative data from clinical studies exist. Nevertheless, the strength of this review is that it likely captures the major themes for clinical decision-making regarding the use of IV BRV in hospital and potentially in the critical care setting.

## Conclusions

In summary, IV BRV shows effectiveness in randomized clinical trials and studies in real-world settings and is generally well tolerated in patients with seizures in the hospital and critical care setting. IV BRV has rapid onset with clinical and electrophysiological improvement in seizures observed within 2 min of administration, minimal drug-drug interactions, a favorable pharmacokinetic profile, and does not require renal dose adjustments. IV BRV represents a clinically relevant ASM for the management of acute seizures in the hospital and potentially in the critical care setting.

## Abbreviations

ASM: antiseizure medication
BID: twice daily
BRV: brivaracetam
ECG: electrocardiogram
EEG: electroencephalography
ICU: intensive care unit
IV: intravenous
LCM: lacosamide
LEV: levetiracetam
MDZ: midazolam
NCSE: nonconvulsive status epilepticus
PET: positron emission tomography
PGTCS: primary generalized tonic-clonic seizures
PPR: photoparoxysmal response
SV2A: synaptic vesicle glycoprotein 2A
TEAE: treatment-emergent adverse event
t_max_: time to maximum plasma concentration
US: United States
VPA: valproate.

## Appendix

**A: Search Method**

**PubMed search**: The following key words were used to search PubMed from inception until April 13, 2021: Primary (base) search string: intravenous AND brivaracetam (33 hits) (https://pubmed.ncbi.nlm.nih.gov/?term=%28%28intravenous%29 + AND + %28brivaracetam%29%29 + AND + %28%28%221996%2F01%2F01%22%5BDate + - + Publication%5D + %3A + %222021%2F04%2F13%22%5BDate + - + Publication%5D%29%29&sort=pubdate). Secondary search strings: brivaracetam AND intravenous AND efficacy (0 new hits); brivaracetam AND intravenous AND safety (0 new hits); brivaracetam and intravenous AND seizure (0 new hits); AND status epilepticus (0 new hits); AND epilepsy monitoring unit (0 new hits); AND intensive care unit (0 new hits); AND critically ill (0 new hits); AND critical care (0 new hits); AND intensive care (0 new hits). Tertiary search strings: brivaracetam and intravenous AND seizure AND critically ill (0 new hits); brivaracetam and intravenous AND seizure AND critically ill AND intensive care (0 new hits).

**American Academy of Neurology 2021 website search**: The following key words were used in the AAN website on April 13, 2021: intravenous brivaracetam (https://index.mirasmart.com/AAN2021/SearchResults.php?q=intravenous + brivaracetam).

**Manual literature search**: Strzelczyk et al. 2017 found through manual literature search of the reference lists of systematic literature reviews and clinical studies.
